# Supplementary material for: RFQAmodel: Random Forest Quality Assessment to identify a predicted protein structure in the correct fold
Source: PLoS One. 2019 Oct 21;14(10):e0218149. doi: 10.1371/journal.pone.0218149 (PMC6802825; doi:10.1371/journal.pone.0218149)
Supplement: S1 File — (PDF) [file pone.0218149.s001.pdf]

Supplementary Information: RFQAmodel: Random Forest Quality Assessment to identify a predicted protein structure in the correct fold.

Clare E. West, Saulo H. P. de Oliveira and Charlotte M. Deane

Contents

A RFQAmodel Random Seed **2**

B Data Sets **2**
B.1 Culling Process 3

C Number of effective sequences **5**

D Prediction of sequence-based descriptors **5**
D.1 Benchmarking secondary structure predictors 5
D.2 Torsion angle prediction 5
D.3 Contact prediction 5
D.4 Fragment library generation 5
D.5 Comparing properties of Training and Validation sets 6

E Estimating the number of models required **8**

F Modelling results **8**

G Comparing Quality Assessment methods **11**

H Model Quality Assessment **12**
H.1 Validation Set 12
H.2 Feature importance 13
H.3 Comparison to a regression model 15
H.4 CASP12 & CASP13 Set 17
H.5 Iterative model generation and model quality assessment 17

A RFQAmodel Random Seed

The random seed was set to 1011 for all RFQAmodel training for reproducibility.

B Data Sets

The mapping between Pfam domains and the PDB structures available on the EBI repository in Feb 2017 encompassed 453,717 protein chains from 113,618 PDB files, mapped to 8,005 unique Pfam families, from which we selected the first protein chain listed for each family as representative.

Table A: Properties of the 8,005 protein chains representing each of the Pfam domains mapped to PDB structures

B_{eff}	Number of protein chains	% of Total	Average length (± s.d.)	Average B_{eff} (± s.d.)
Less than 100	1,948	24.33	124 ± 121	30 ± 28
100 to 1,000	3,138	39.2	138 ± 112	429 ± 244
More than 1,000	2,919	36.46	164 ± 118	5,470 ± 8,094

Table B: Properties of the 4,728 protein chains with SCOPe annotations chosen to represent unique Pfam families mapped to PDB structures

B_{eff}	Number of protein chains	% of Total<br>%	Average length (± s.d.)	Average B_{eff} (± s.d.)
Less than 100	918	19.41	124 ± 124	29 ± 28
100 to 1,000	1,498	31.68	129 ± 95	452 ± 254
More than 1,000	2,312	48.90	159 ± 112	6,030 ± 8,643

Table C: Properties of the 488 protein domains chosen to comprise our Training and Validation data sets.

B_{eff}	Number of protein chains	% of total	Average length (± s.d.)	Average B_{eff} (± s.d.)
Less than 100	76	25	124± 48	41± 31
100 to 1,000	164	55	134±53	455± 262
More than 1,000	248	83	136± 51	6924± 8867

B.1 Culling Process

We removed protein chains from the set of 4,728 proteins in Table 2 according to the following criteria:

- Remove all protein chains shorter than 50 residues (4,060 protein chains remaining).
- Remove all protein chains that are classified in SCOP as multi-domain, membrane, or small protein (3,688 protein chains remaining).
- Remove all non-crystallographic structures and those with a resolution of 2.5Å or worse (2,266 protein chains remaining).
- Remove all protein chains that contain more than one domain as annotated on the PDB mapping to Pfam (1,376 single-domain protein chains remaining).
- Remove all proteins that could not be parsed using the non-permissive parser of BioPython [1] (1,327 single-domain protein chains remaining). Extract the corresponding domain as annotated on the mapping.
- Remove all protein domains with chain breaks, incomplete sequences, and unresolved residues (606 protein domains remaining).
- Remove all protein domains longer than 250 residues (488 protein domains remaining). For this step, one additional protein domain was discarded as its PDB contained only Cα coordinates.

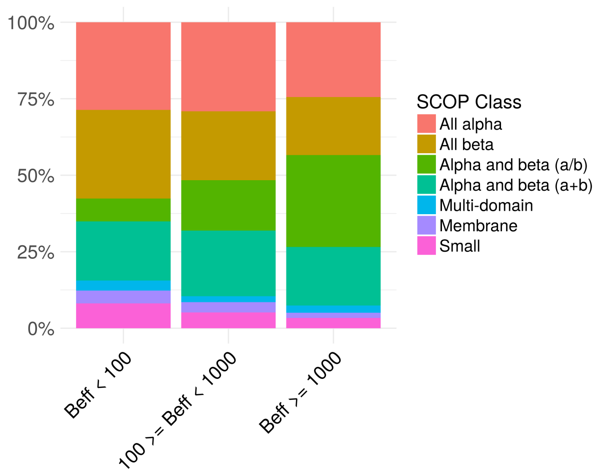

Figure A: **SCOP classes of representative chains**
Proportion of protein chains per SCOP class at different B_{eff} levels. Data is shown for the 4,728 protein chains with SCOP annotations chosen to represent unique families described on the PDB mapping to Pfam.

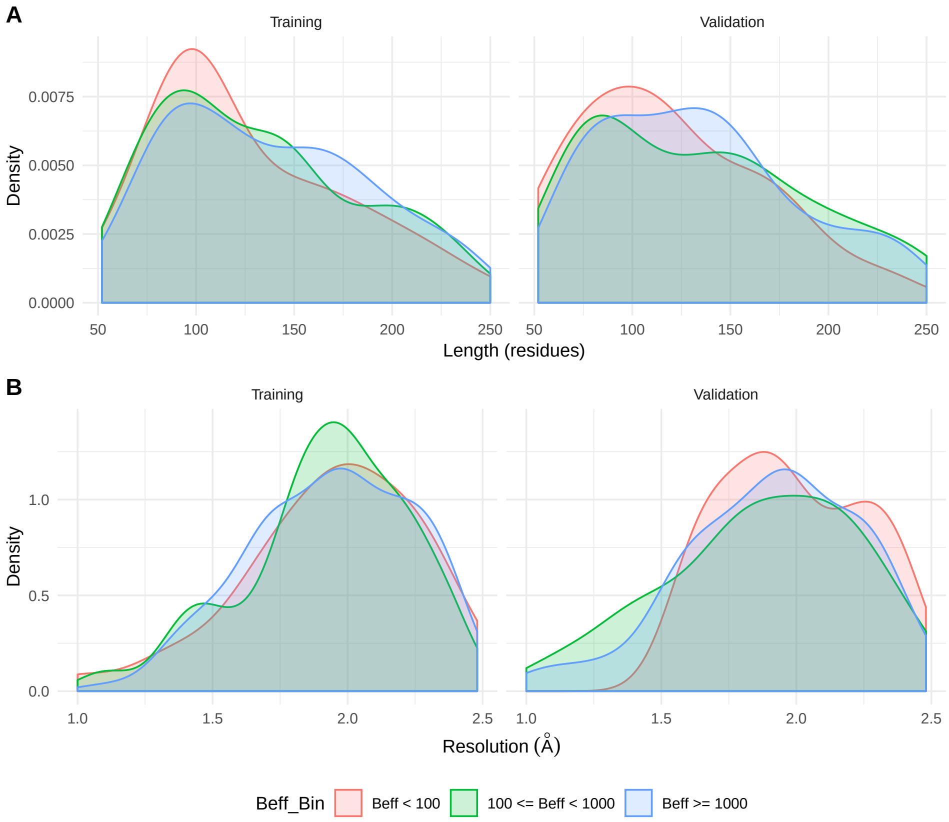

Figure B: **Domain lengths and resolutions of Training and Validation sets.**
Distribution of the A) lengths and B) resolution of protein domains in our Training (left) and Validation (right) sets according to three B_{eff} bins.

C Number of effective sequences

The number of effective sequences [2] is defined as:

$$B_{\text{eff}} = \sum_{b=1}^{B} \frac{1}{m_b} \qquad (1)$$

Where m_b is the number of sequences in the multiple sequence alignment with at least 90% sequence identity to the b-th sequence in the alignment, and B is the total number of sequences.

D Prediction of sequence-based descriptors

To perform protein structure prediction, we computed several sequence-based descriptors that are commonly used as input for modelling. We will describe the prediction of these descriptors in detail over the next sections.

D.1 Benchmarking secondary structure predictors

Secondary structure prediction is an essential step in most template-free protein structure prediction protocols. Errors in secondary structure prediction can have a drastic effect on modelling success, particularly if a large stretch of residues are assigned incorrectly. Recently, two methods, SPIDER3 [3], and DeepCNF [4], have used deep learning to improve the precision of secondary structure prediction. Given the importance of secondary structure prediction to accurate modelling, we compared the Q3 and Q8 precisions of three secondary structure predictors, PSIPRED [5], SPIDER3 [3], and DeepCNF [4] (Figure C). To perform this assessment, we considered the methods' ability to classify residues into three (helical, strand, or coil) or eight secondary structure types (refer to [6] for more details), respectively.

For B_{eff} <1000, no significant difference was observed when assessing the Q3 precision obtained by each of the methods. However, DeepCNF produced better Q3 predictions when sufficient sequence information was available (B_{eff} ≥1,000). DeepCNF produced better Q8 predictions than the other methods for all B_{eff} bins. Our subsequent analyses were carried out using the output of DeepCNF Q8.

D.2 Torsion angle prediction

We used SPIDER3 [3] with standard parameters to perform torsion angle prediction for the 488 protein domains comprising our Training and Validation sets. To validate torsion angle predictions, we used BioPython to extract the torsion angles from the native structure of each domain.

D.3 Contact prediction

Contact prediction was carried out using metaPSICOV [7] with standard parameters for the 244 proteins in each of our Training and Validation sets. MetaPSICOV uses a two-stage neural network that outputs two sets of predictions (stage1 and stage2). Although metaPSICOV stage2 predictions have been shown to be more precise than stage1, it has been reported that predictions from stage1 lead to better modelling results [7, 8]. For this reason, we have opted to use metaPSICOV stage1 predictions.

D.4 Fragment library generation

Flib-Coevo was used to produce fragment libraries for all proteins in our Training and Validation data sets. DeepCNF Q8 secondary structure predictions and otherwise standard parameters were used. Fragments from homologues were discarded to represent a more realistic template-free protein structure prediction scenario.

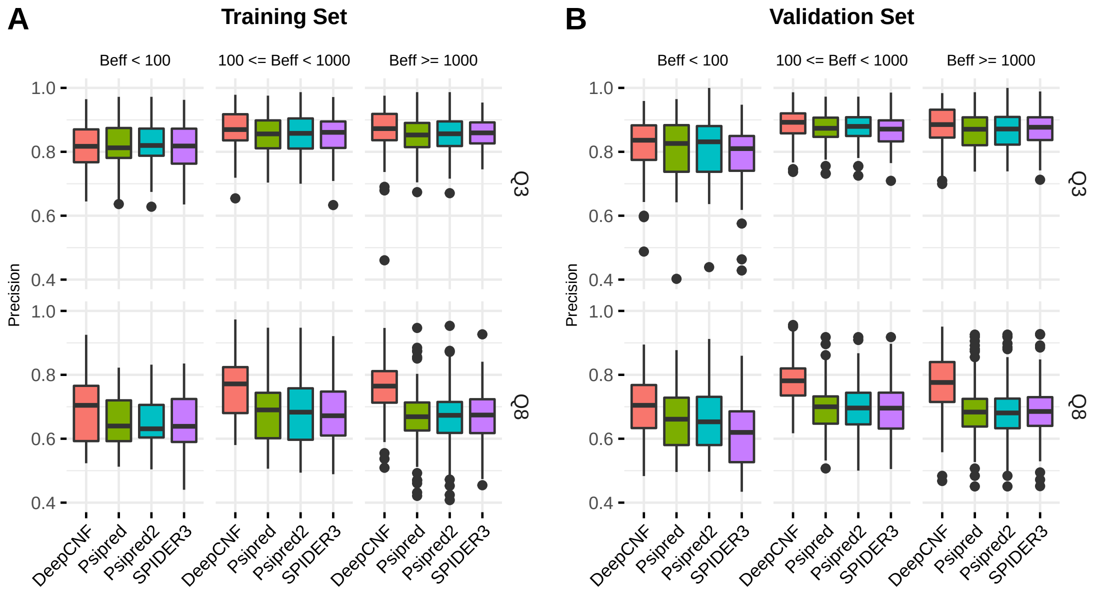

Figure C: **Secondary structure prediction.**
Precision of Q3 (top) and Q8 (bottom) secondary structure prediction according to B_{eff} for the 244 targets in each of the **A**) Training set and **B**) Validation set.

D.5 Comparing properties of Training and Validation sets

To perform classification, it is ideal to train and validate on well-balanced sets with similar properties. Unbalanced sets may lead to overtraining or underestimation of the classifier's performance. For that reason, we built our Training and Validation sets to ensure that both sets were well balanced in terms of several properties known to have an impact on modelling success.

As described in the previous section, secondary structure prediction can have an impact on modelling success. We compared the Q3 and Q8 precisions of secondary structure prediction on our Training and Validation sets (Figure C) and found comparable Q3 and Q8 precisions between the two sets.

We then compared the precision of Torsion Angle prediction between our Training and Validation sets. In our current modelling protocol, Torsion Angle prediction is used for building a fragment library. Previous findings suggest that poor torsion angle prediction leads to modelling failure [9]. To perform this

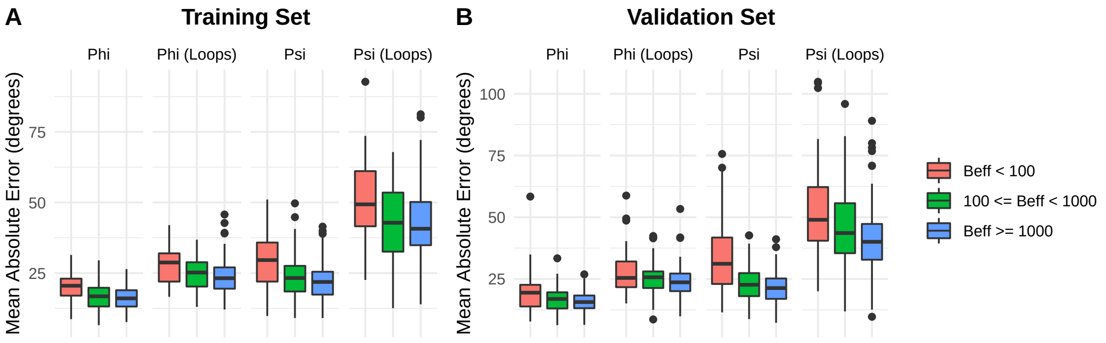

Figure D: **Torsion angle prediction.**
Mean Absolute Error (MAE) of Φ and Ψ torsion angle prediction for the 244 proteins in each of the A) Training set and B) Validation set (right). MAE was calculated across all residues or across loop residues exclusively.

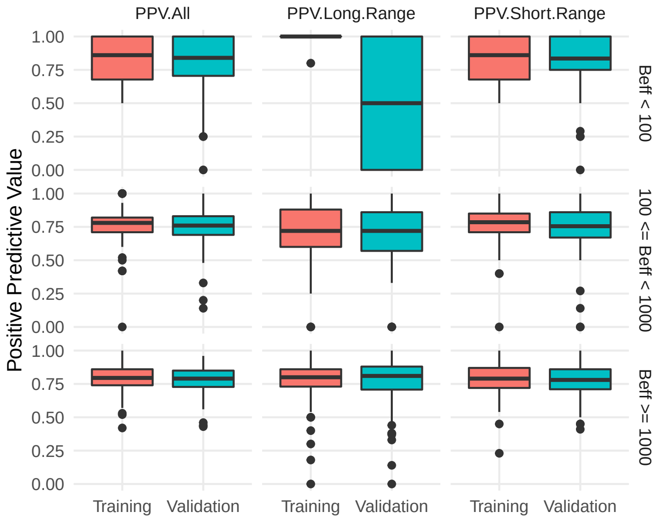

Figure E: **Contact prediction.**
Precision (Positive Predictive Value, PPV) of short-range (<23 residues separation) and long-range (≥23 residue-separation) predicted contacts as output by metaPSICOV stage1 for the 244 protein domains in our Training set and the 244 protein domains in our Validation set. Results are shown for three different B_{eff} bins.

comparison, we calculated the Mean Absolute Error (MAE) in degrees between predicted and observed angles for both Φ and Ψ dihedrals, across all domains in our Training and Validation sets (Figure D). Considering the challenges associated with predicting loop conformations during modelling [9], we have also assessed the MAE exclusively for loop residues of these domains. For this, loop residues were determined using the DSSP secondary structure assignments calculated previously.

Our results reveal that the precision of torsion angle prediction is slightly lower for lower B_{eff} values. Interestingly, the MAE for Ψ angles was significantly higher than for Φ angles. The difference in Φ against Ψ MAEs becomes significant when we considered the MAE for loop residues, exclusively. Comparable MAEs were observed between Training and Validation sets for both Φ and Ψ angles, considering all residues and considering loops exclusively.

Another property that has been shown to be crucial for modelling success [8] is the precision of contacts predicted from multiple sequence alignments. To ensure that our split between Training and Validation was balanced in this regard, we compared the precision of short-range (<23 residues separation), and long-range (≥ 23 residues separation) predicted contacts output by metaPSICOV stage1 for all proteins in our Training and Validation sets (Figure E). The choice of stratifying at 23 residues separation is corroborated by previous findings suggesting that precision of long-range contacts is more important than short-range for modelling success [7, 8]. Our analysis shows that the two data sets are comparable both in terms of precision of short-range and long-range predicted contacts across each of three B_{eff} bins.

E Estimating the number of models required

First, we used SAINT2 to produce 1,000 models per target for 245 targets randomly selected from our Training and Validation sets. For each target, we sampled n models without replacement and counted the number of targets for which at least one correct model (TM-score ≥ 0.5) was included in the sample for varying values of n ($1 \leq n \leq 1,000$) (Figure F). We observed little improvement in the number of correctly predicted targets when sampling more than 500 models. To balance the number of modelling successes and computational feasibility, we therefore opted to produce 500 models using SAINT2 for our subsequent analyses.

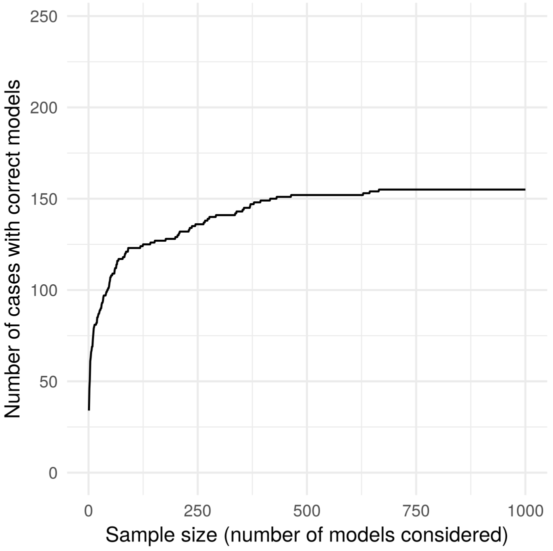

Figure F: The number of targets with at least one correct model (TM-Score ≥ 0.5) in a sample of all models produced by SAINT2. The sample size ranges from 1 to 1,000 models considered per target. Results are shown for 245 protein domains randomly selected from our Training and Validation sets.

F Modelling results

Our modelling results vary by B_{eff}, SCOP class and domain length. For our Training set, we observe a modelling success rate of 46% at B_{eff} values below 100, and a success rate of 69% for $B_{\text{eff}} \geq 1000$ (Fig GA). SAINT2 produced a correct model for 85% of all-α targets, 65% of α/β targets, 61% of α+β targets, and 30% of all-β targets (Fig GB). As expected, modelling success rate decreases as targets increase in length (Fig GC). For our Training set, SAINT2 produced a correct model for 83% of the targets that were 50 to 99 residues-long, for 65% of targets that were 100 to 149 residues-long, for 41% of targets that were 150 to 199 residues-long, and 39% of targets longer than 200 residues.

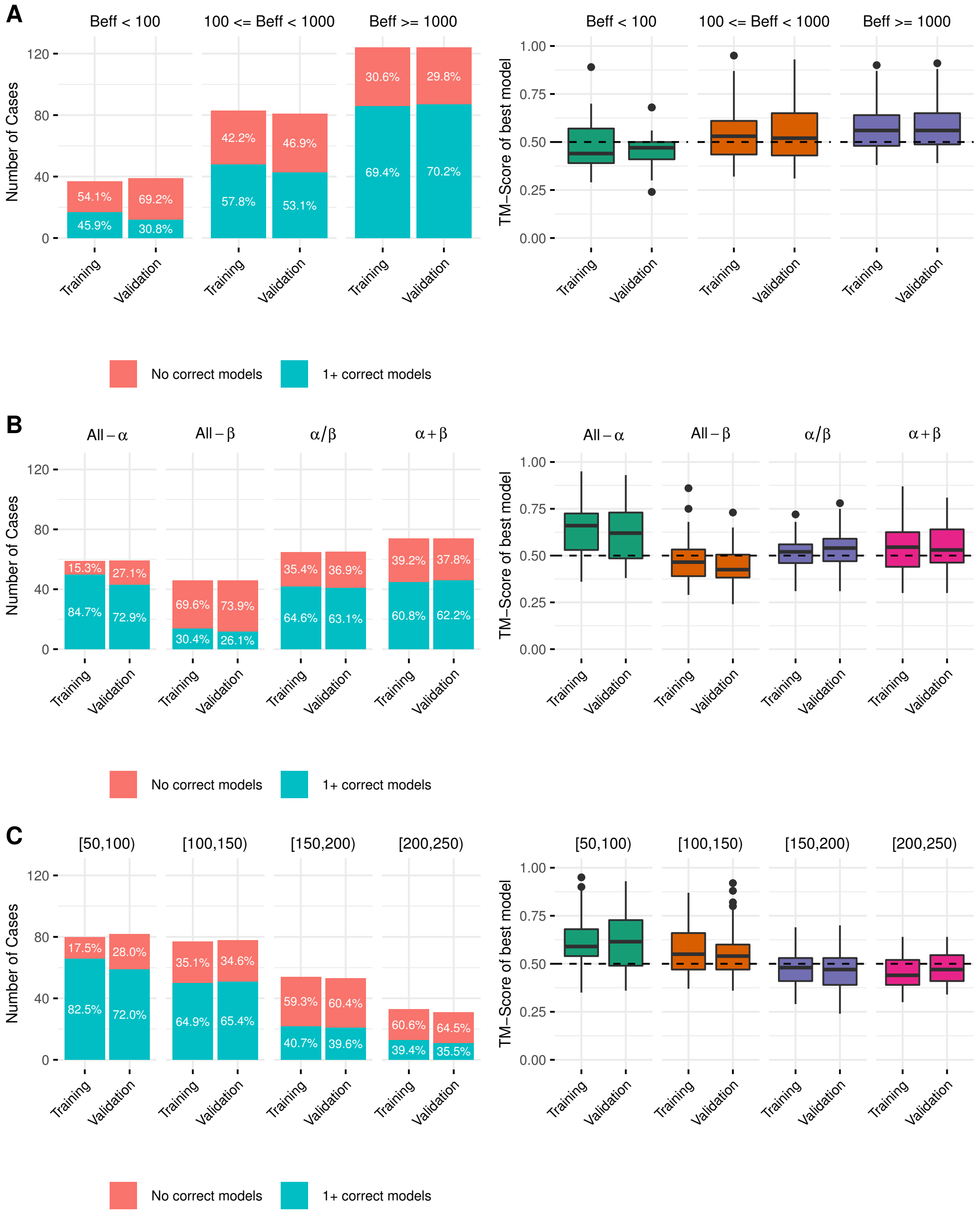

Figure G: **Modelling success rate by B_{eff}, SCOP class, and domain length.**
Number of targets for which correct models were (blue) or were not (red) produced (left) and distributions of the TM-score of best model (right) for the 244 protein domains in each of our Training and Validation sets. Results are shown for different **A**) B_{eff} bins **B**) SCOP class and **C**) domain length. A model with a TM-score ≥ 0.5 is considered correct.

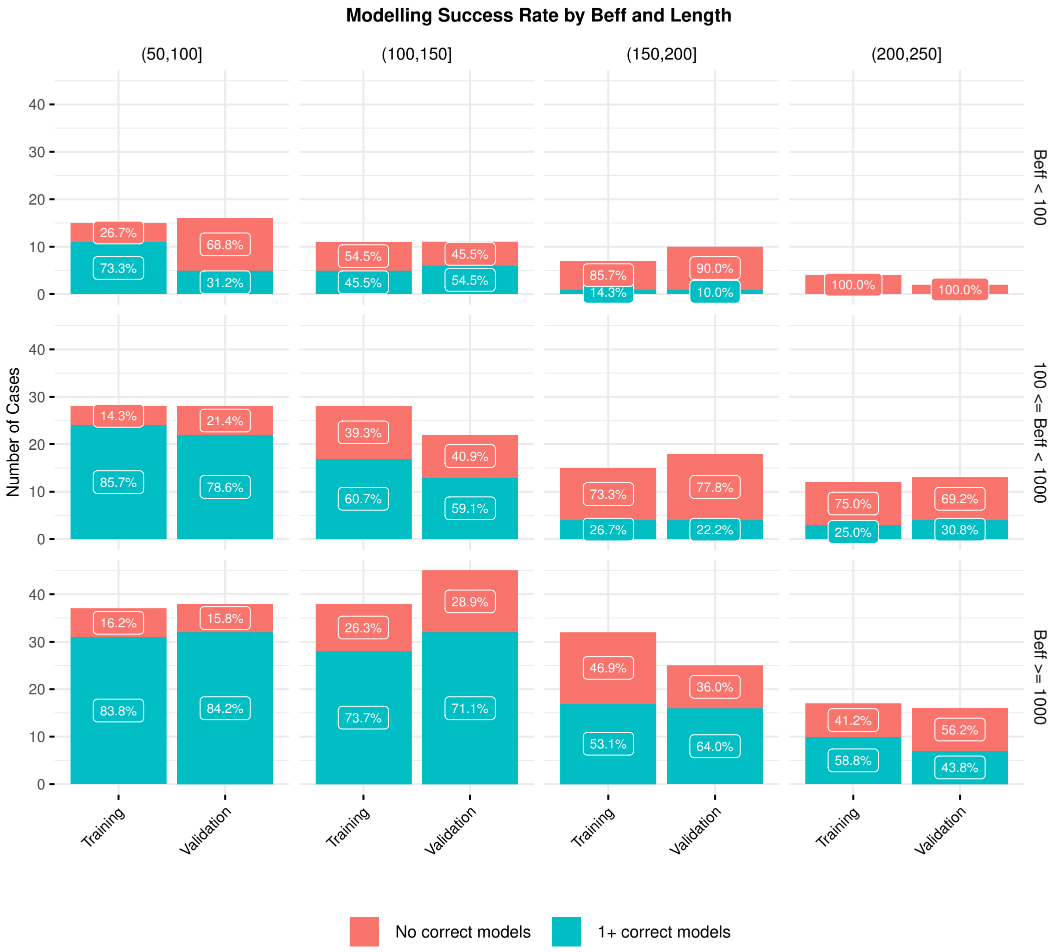

Figure H: **Modelling success rate by both B_{eff} and length.**
Modelling success rate for the 244 protein domains in each of our Training and Validation sets according to both length and B_{eff} bins. Any target for which at least one correct model (TM-Score ≥ 0.5) was produced is considered a modelling success.

G Comparing Quality Assessment methods

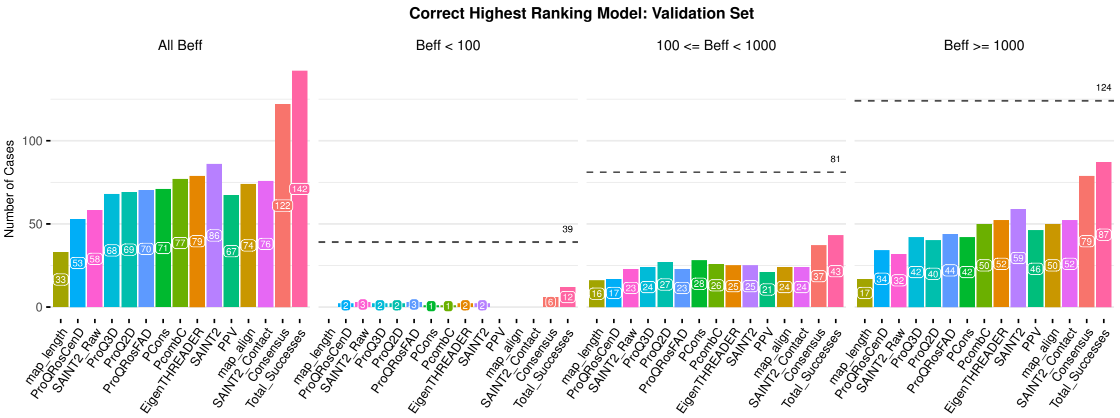

Figure I: **Ranking of models for Validation Set targets**
The number of targets out of the 244 targets in our Validation set for which a correct model was produced and selected as the highest-ranked model according to 13 methods. Three SAINT2 scores (SAINT2, SAINT2_Contact and SAINT2_Raw), seven existing quality assessment scores (ProQ3D, ProQRosCenD, ProQRosFAD, Pcons, PcombC, ProQ2D and PPV), and three predicted contact map alignment scores (EigenTHREADER, Map_align and map_length) are shown, as well as all methods combined ("Consensus") and the total number of targets with a correct model ("Total Successes"), for three B_{eff} bins and across all bins. The total number of targets in each B_{eff} bin is indicated with a dashed line.

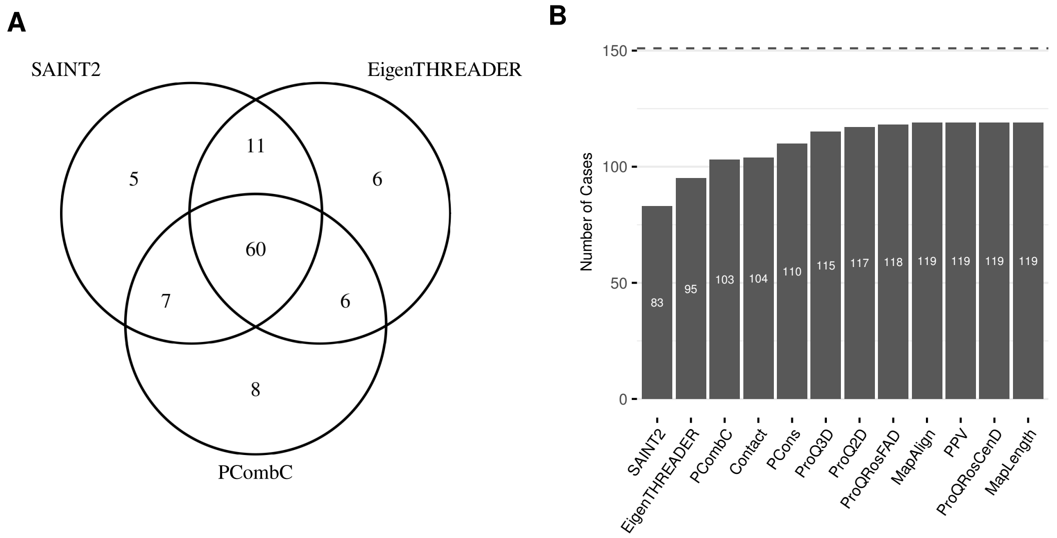

Figure J: **A**) The number of targets in our Training Set for which the highest-ranking model is correct, when ranked according to the three overall best methods: the SAINT2 score, quality assessment score PcombC, and predicted contact alignment score EigenTHREADER. **B**) The number of targets for which at least one method identified a correct model as the highest-ranking, when methods are sequentially added in order of ranking ability as shown in Figure A (from left to right). The total number of targets with at least one correct model is indicated with a dashed line.

H Model Quality Assessment

H.1 Validation Set

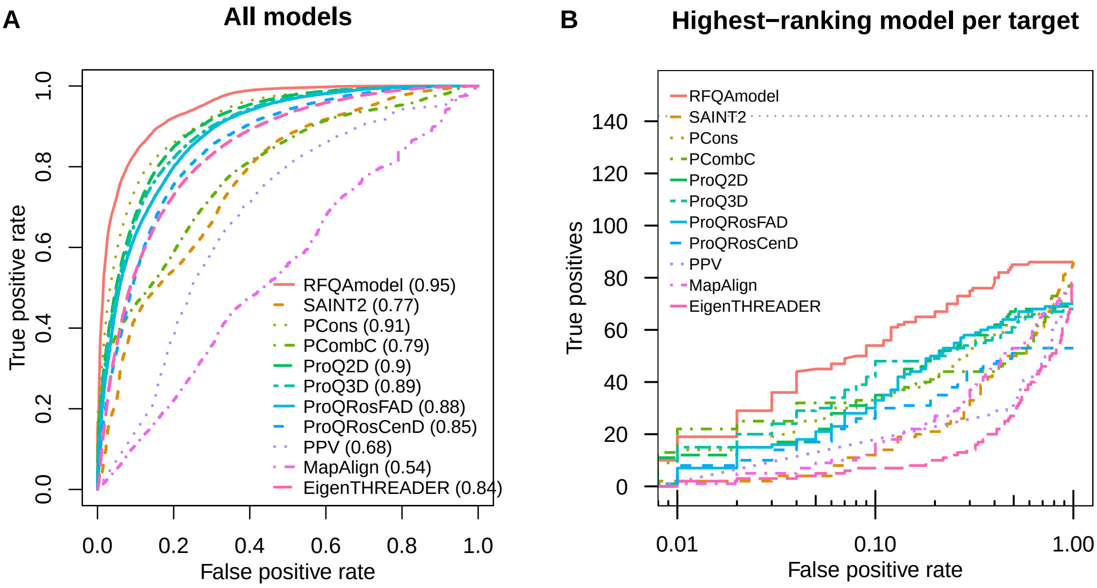

Figure K: **Classification of Validation Set targets.**
A) Receiver Operating Characteristic (ROC) Curves for the classification of all models into whether they were correct (TM-score ≥ 0.5) or incorrect according to RFQAmodel and the 10 component methods for the 244 targets in our Validation set. The area under the ROC curve (AUC) for each method is shown in brackets. The EigenTHREADER score was normalised by the maximum score for each target. B) The number of targets with a correct highest-ranking model (true positives) plotted against the false positive rate on a logarithmic scale. The grey dotted line indicates the total number of targets for which at least one correct model was produced by SAINT2.

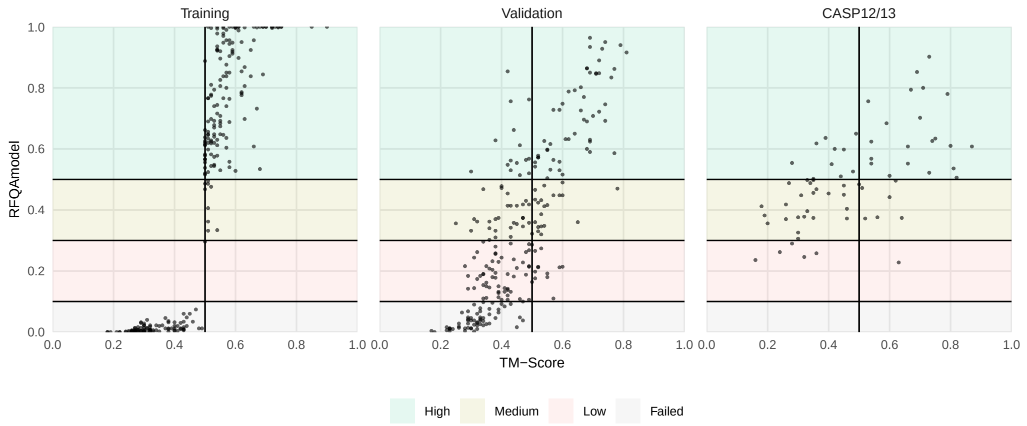

Figure L: The RFQAmodel score and TM-score of the highest-ranking model per target in the Training and Validation sets, as well as the CASP12 and CASP13 targets. Predictions are categorised into high (>0.5, green), medium (between 0.3 and 0.5, yellow), or low (between 0.1 and 0.3, red) confidence, or are predicted to have failed (≤ 0.1, grey).

H.2 Feature importance

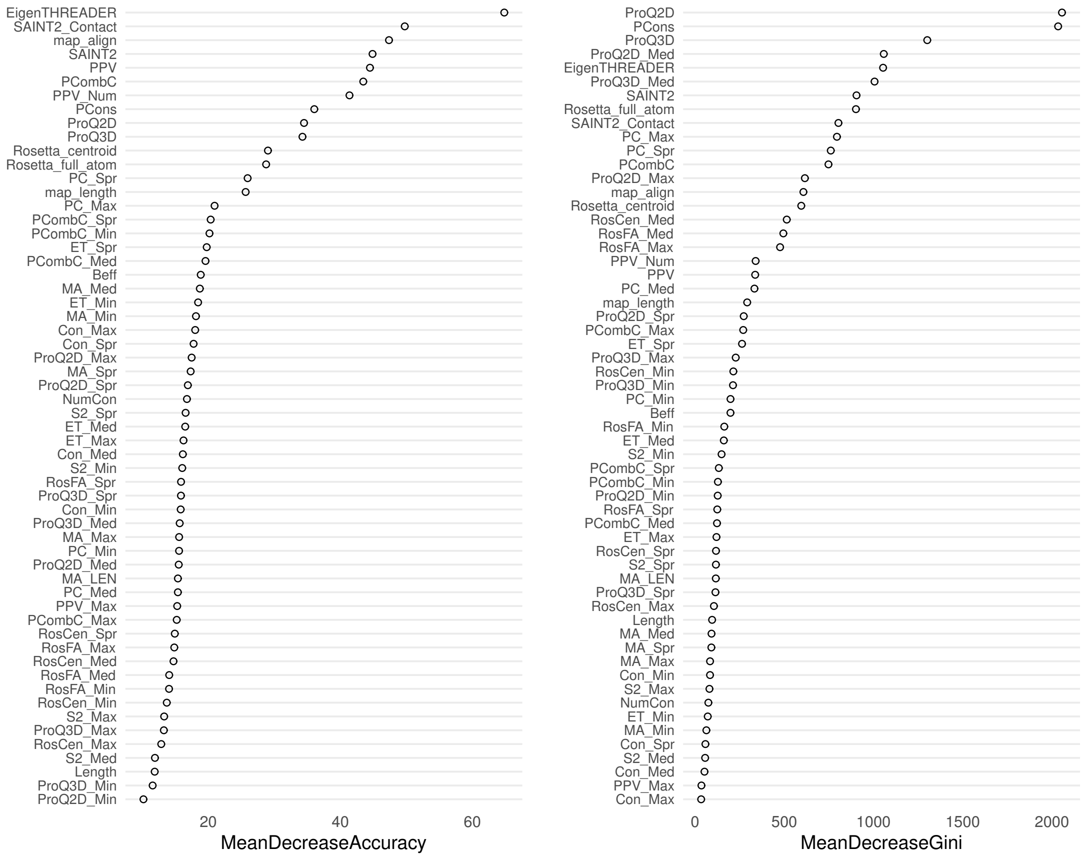

Figure M: **Relative importance of the component features of RFQAmodel reported by randomForest.**

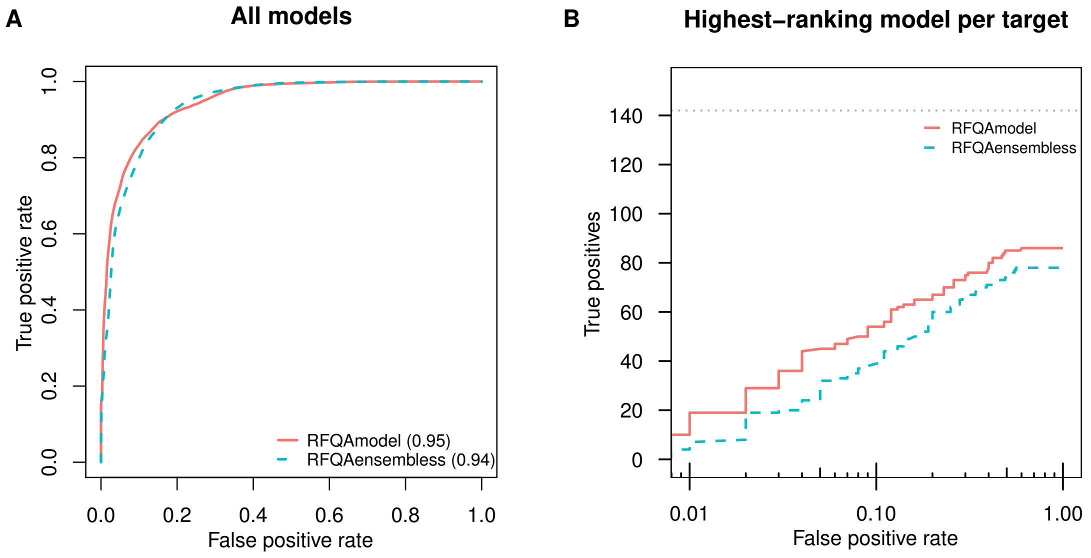

Figure N: **Classification of Validation Set targets when ensemble features are excluded.**
A) Receiver Operating Characteristic (ROC) Curves for the classification of all models into whether they were correct (TM-score ≥ 0.5) or incorrect according to RFQAmodel and a version of the model trained without the ensemble features (RFQAensembless). The area under the ROC curve (AUC) for each method is shown in brackets. B) The number of targets with a correct highest-ranking model (true positives) plotted against the false positive rate on a logarithmic scale. The grey dotted line indicates the total number of targets for which at least one correct model was produced by SAINT2.

H.3 Comparison to a regression model

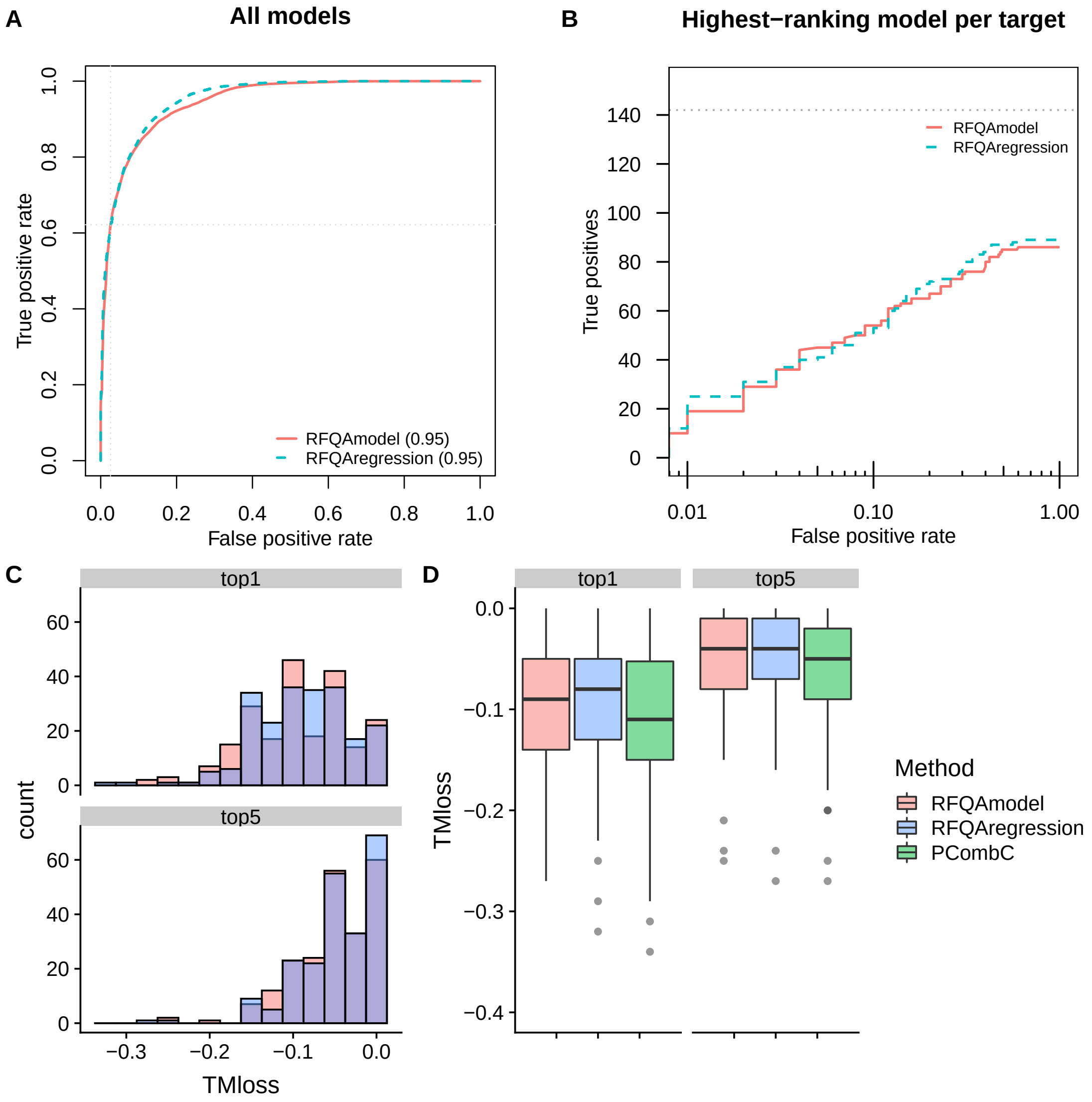

Figure O: **Performance of RFQAmodel compared to a version of the model trained to predict the actual TMscore of each model (RFQAregression)**

A) Receiver Operating Characteristic (ROC) Curves for the classification of all models into whether they were correct (TM-score ≥ 0.5) or incorrect according to RFQAmodel and RFQAregression. The area under the ROC curve (AUC) for each method is shown in brackets. The grey dotted lines indicate the performance when a cutoff of 0.5 is used. B) The number of targets with a correct highest-ranking model (true positives) plotted against the false positive rate on a logarithmic scale. The grey dotted line indicates the total number of targets for which at least one correct model was produced by SAINT2. C) The distribution of TMloss - the difference between the TM-score of the highest-ranking model (top1) or the best of the top five highest ranking model (top5) and the best model in the ensemble - for RFQAmodel and RFQAregression. Results are shown for all targets with at least one model with a TM-score above 0.4. D) The distributions of TMloss for RFQAmodel and RFQAregression compared to the component method PcombC.

Table D: **Performance on the Validation Set for RFQAmodel compared to RFQAregression, which is trained to predict the actual TMscore for each model**. The results are divided into confidence categories for RFQAmodel, or predicted TMscore range for RFQAregression, as well as for all targets overall (All). The total number of targets (Total) and the number of targets for which there is at least one correct model in the ensemble (Max) are shown for each category. The number of targets for which the highest-ranking model (Top1) or the best of the top five highest-ranking models (Top5) is correct is shown, with the corresponding precision in brackets.

	Confidence	Total	Max	Top1		Top5	
RFQAmodel	High	67	63	52	(77.6%)	60	(89.6%)
	Medium	50	38	21	(42.0%)	30	(60.0%)
	Low	68	36	13	(19.1%)	21	(30.9%)
	Failed	59	5	0	(0.0%)	1	(1.7%)
	All	244	142	86	(35.2%)	112	(45.9%)
RFQAregression	≥ 0.5	57	54	46	(80.7%)	52	(91.2%)
	(0.3, 0.5)	171	88	43	(25.1%)	65	(38.0%)
	(0.1, 0.3)	16	0	0	(0.0%)	0	(0.0%)
	All	244	142	89	(36.5%)	117	(48.0%)

H.4 CASP12 & CASP13 Set

Table E: **RFQAmodel performance for all CASP12 and CASP13 free-modelling and template-based modelling targets.** Targets are grouped into free-modelling (FM) and template-based modelling (TBM) targets. For each RFQAmodel confidence category, we report: the total number of targets (Total); the number of targets for which there was a correct model among the 500 models (Max); and the number of targets for which the highest-ranking model (Top1) or best of the top five highest-ranking models (Top5) is correct with the corresponding precision in brackets.

	Type	Confidence	Total	Max	Top1		Top5	
CASP12	FM	High	9	8	5	(55.6%)	7	(77.8%)
		Medium	20	11	6	(30.0%)	6	(30.0%)
		Low	4	3	1	(25.0%)	1	(25.0%)
		All	33	22	12	(36.4%)	14	(42.4%)
	TBM	High	23	23	23	(100.0%)	23	(100.0%)
		Medium	1	1	1	(100.0%)	1	(100.0%)
		All	24	24	24	(100.0%)	24	(100.0%)
CASP13	FM	High	22	21	16	(72.7%)	20	(90.9%)
		Medium	10	4	1	(10.0%)	3	(30.0%)
		Low	2	0	0	(0.0%)	0	(0.0%)
		All	34	25	17	(50.0%)	23	(67.6%)
	TBM	High	36	35	31	(86.1%)	33	(91.7%)
		Medium	1	1	1	(100.0%)	1	(100.0%)
		All	37	36	32	(86.5%)	34	(91.9%)

H.5 Iterative model generation and model quality assessment

Table F: **Performance of iterative RFQAmodel on the 50 targets categorised as medium confidence**. Results are shown for the original ensembles of 500 models, the ensembles when models are generated until either a model is categorised as high confidence or 10,000 models have been generated (Iterative RFQAmodel), and for ensembles of 10,000 models for all targets. The total number of targets (Total) as well as the number of targets with at least one correct model is reported for each confidence category (Confidence) and for all targets overall (All). The number of targets for which the highest-ranking model (Top1) or the best of the top five highest-ranking models (Top5) is correct is shown, with the corresponding precision in brackets.

	Confidence	Total	Max	Top1		Top5	
500 models	Medium	50	38	21	(42.0%)	30	(60.0%)
Iterative RFQAmodel	High	36	34	19	(52.8%)	28	(77.8%)
	Medium	14	11	3	(21.4%)	5	(35.7%)
	All	50	45	22	(44.0%)	33	(66.0%)
10000 models	High	34	34	22	(64.7%)	25	(73.5%)
	Medium	16	13	4	(25.0%)	7	(43.8%)
	All	50	47	26	(52.0%)	32	(64.%)0

References

[1] Cock PJA, Antao T, Chang JT, Chapman BA, Cox CJ, Dalke A, et al. Biopython: Freely available Python tools for computational molecular biology and bioinformatics. Bioinformatics. 2009;25(11):1422–1423. doi:10.1093/bioinformatics/btp163.

[2] Michel M, Skwark MJ, Hurtado DM, Ekeberg M, Elofsson A. Predicting accurate contacts in thousands of Pfam domain families using PconsC3. Bioinformatics. 2017;doi:10.1093/bioinformatics/btx332.

[3] Heffernan R, Yang Y, Paliwal K, Zhou Y. Capturing non-local interactions by long short-term memory bidirectional recurrent neural networks for improving prediction of protein secondary structure, backbone angles, contact numbers and solvent accessibility. Bioinformatics. 2017;doi:10.1093/bioinformatics/btx218.

[4] Wang S, Peng J, Ma J, Xu J. Protein Secondary Structure Prediction Using Deep Convolutional Neural Fields. Sci Rep. 2016;6(1):18962. doi:10.1038/srep18962.

[5] Jones DT. Protein secondary structure prediction based on position-specific scoring matrices 1 1Edited by G. Von Heijne. J Mol Biol. 1999;292(2):195–202. doi:10.1006/jmbi.1999.3091.

[6] Kabsch W, Sander C. Dictionary of protein secondary structure: Pattern recognition of hydrogen-bonded and geometrical features. Biopolymers. 1983;22(12):2577–2637. doi:10.1002/bip.360221211.

[7] Jones DT, Singh T, Kosciolek T, Tetchner S. MetaPSICOV: Combining coevolution methods for accurate prediction of contacts and long range hydrogen bonding in proteins. Bioinformatics. 2015;31(7):999–1006. doi:10.1093/bioinformatics/btu791.

[8] de Oliveira SHP, Shi J, Deane CM. Comparing co-evolution methods and their application to template-free protein structure prediction. Bioinformatics. 2016;33(3):btw618. doi:10.1093/bioinformatics/btw618.

[9] de Oliveira SHP, Shi J, Deane CM. Building a better fragment library for de novo protein structure prediction. PLoS One. 2015;10(4):1–20. doi:10.1371/journal.pone.0123998.
